# An Overview of Experimental and Clinical Spinal Cord Findings in Alzheimer’s Disease

**DOI:** 10.3390/brainsci9070168

**Published:** 2019-07-17

**Authors:** Qing Xie, Wei-Jiang Zhao, Guan-Yong Ou, Wei-Kang Xue

**Affiliations:** Center for Neuroscience, Shantou University Medical College, 22 Xin Ling Road, Shantou, Guangdong 515041, China

**Keywords:** Alzheimer’s disease (AD), spinal cord, pathological changes, β-amyloid protein, tau protein, inflammation

## Abstract

Alzheimer’s disease (AD) is a neurodegenerative disorder that occurs mainly in the elderly and presenile life stages. It is estimated that by the year 2050, 135 million people will be affected by AD worldwide, representing a huge burden to society. The pathological hallmarks of AD mainly include intracellular neurofibrillary tangles (NFTs) caused by hyperphosphorylation of tau protein, formation of extracellular amyloid plaques, and massive neural cell death in the affected nervous system. The pathogenesis of AD is very complicated, and recent scientific research on AD is mainly concentrated on the cortex and hippocampus. Although the spinal cord is a pivotal part of the central nervous system, there are a limited number of studies focusing on the spinal cord. As an extension of the brain, the spinal cord functions as the bridge between the brain and various parts of the body. However, pathological changes in the spinal cord in AD have not been comprehensively and systematically studied at present. We here review the existing progress on the pathological features of AD in the spinal cord.

## 1. Introduction

Alzheimer’s disease (AD) is a neurodegenerative disorder of the central nervous system, with the principal clinical symptoms being disorders of recent memory. According to epidemiological survey data, 62% of dementia cases worldwide had occurred in developing countries (low- and middle-income countries) as of 2013, and by the year of 2050, developing countries will possess 71% of all dementia cases [1], with rapid increases in number expected to occur in China, India, and some other countries in the south of Asia and Western Pacific. In 2010, Alzheimer’s Disease International (ADI) reported that the annual cost of treatment and care for dementia will exceed $604 billion worldwide in the current absence of any effective way to cure or control the disease progression [2]. AD has been considered as one of the most lethal diseases, with the fourth highest mortality, following cardiovascular and cerebrovascular diseases, tumors, and stroke, making the prevention and treatment of AD very important and urgent [1,2].

The etiology and pathogenesis of AD are very complex, and there have been a variety of theories, the most popularly accepted being gene mutation [3], Aβ toxicity [4], abnormal tau protein modification, oxidative stress [5] and inflammation [6], as well as cholinergic neuronal damage and neurovascular theories [7,8]. However, no specific and authoritative conclusions have been achieved. The main pathological features of AD include the formation of extracellular amyloid plaque deposits, intracellular neurofibrillary tangles (NFTs) formed by hyperphosphorylation of tau protein, and a large amount of neuronal cell death and synaptic changes [4,5].

At present, research on AD is mostly focused on brain tissue, whereas other parts of the CNS, including the spinal cord, are less studied. As an extension of the brain, the spinal cord shares the same cellular components with many brain regions and is responsible for transmitting the motor nerve signals from the brain to the body’s trunk and limbs. At the same time, the spinal cord conveys sensory information from various parts of the body to the brain [9]. Reduced choline acetyltransferase activity was found in both anterior and posterior gray of the spinal cord, suggesting that it can be pathologically affected in Alzheimer-type dementia [10]. In addition, the spinal cord is simpler in terms of cell architecture and neuronal fiber connectivity, and these unique characteristics can facilitate us in deeply understanding the pathophysiological progression of AD in other brain regions. 

It has been recently reported that the cumulative incidence of AD in patients with spinal cord injury (SCI) is higher than in non-SCI patients, with a higher hazard ratio than in non-SCI patients, suggesting that SCI patients have an increased risk of AD development [11]. Frequent and precocious impairment of gait possibly related to the pathology of the spinal cord has been reported [9]. This article summarizes existing research on the pathological features of AD in the spinal cord, which may facilitate further understanding of the development of AD and relevant clinical management.

## 2. Abnormal Phosphorylation of Tau in the Spinal Cord of AD Patients and Animal Models

The tau protein is a hydrophilic, soluble, and nonfolded microtubule-associated protein (MAP) under normal conditions. It functions to stabilize neuron microtubules and plays a major role in maintaining neuronal growth, polarity, and intracellular substance transport [12]. A large number of studies have indicated that tau plays an important role in promoting the development of AD [13,14,15].

Yamada failed to detect NFTs in the spinal cord of AD patients but succeeded in identifying a small number of NFTs in cell groups from the intermediolateral, posterior, anterolateral, and anteromedian zones of the spinal cord from senile dementia patients [16]. However, research by Saito [17] demonstrated tau immunoreactivity in neurons of the anterior horn in all AD cases, with a lesser extent detected in the intermediate zone and in the posterior horn. NFTs were identified in most AD cases investigated. Research by Dugger et al. [18] showed by immunohistochemical staining that hyperphosphorylation of tau is very common in the spinal cord of AD patients and normal elderly. Their studies involved 46 cases of patients clinically diagnosed with AD, among which 96% showed tau phosphorylation in the cervical spinal segment, 69% showed tau phosphorylation in the thoracic spinal segment, 65% showed tau phosphorylation in the lumbar spinal segment, and 53% showed tau phosphorylation in the sacral spinal segment [18]. In coronal sections of the spinal cord, NFTs were particularly obvious in the ventral horn. In the meantime, deposition of hyperphosphorylated tau was also sparsely detected in each segment of the spinal cord in 37 cases of nondementia patients with no diagnosis of any central nervous system diseases, indicating that accumulation of hyperphosphorylated tau may occur before dementia occurrence, and that hyperphosphorylation of tau may occur in the spinal cord in the preclinical phase of AD [18]. Zhu et al. [19] reported a few argentophilic, tau-positive NFTs and neuropil threads in the anterior horn at both cervical and lumbar segments of the spinal cord from AD patients.

**Figure 1:**
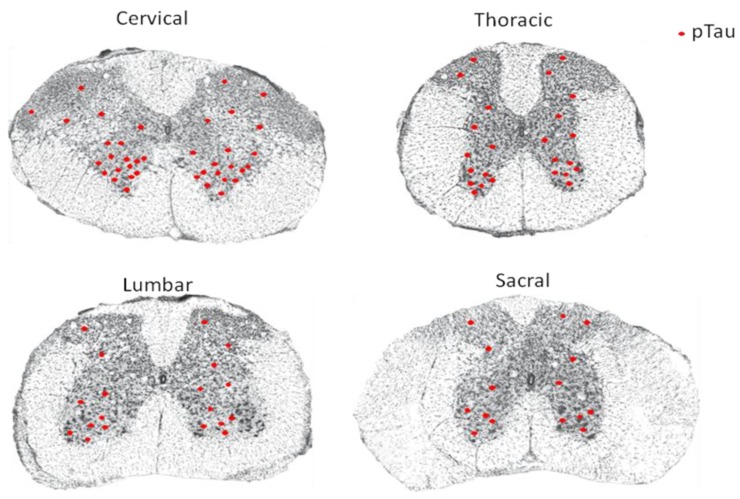
Schematic diagram summarizing the distribution of hyperphosphorylated tau protein (pTau) in each segment of the spinal cord from Alzheimer’s disease (AD) patients based on descriptions from References [13,14,15,16,17,18,19,20,21,22,23,24,25]. The red dot indicates pTau.

In agreement with observations in both Tg30tau and P301L transgenic mice [20,21], other studies demonstrated hyperphosphorylation of tau in ventral horn neurons and glial cells, and neuronal axons in the spinal cord of AD patients, and the severity of tau phosphorylation is correlated with that of NFT in the brain [22,23]. Research in patients being affected by neurodegenerative diseases in the United States also came to the same conclusion that NFTs exist in the spinal cord of all the AD patients. Immunohistochemical staining suggested that NFTs are formed by hyperphosphorylated tau protein [24,25]. NFTs caused by hyperphosphorylation of tau protein were found in each segment of the spinal cord in AD patients, and the degree of tangles was more prominent in the ventral horn of the spinal cord (Figure 1). 

Intracellular NFTs are mainly composed of paired helical filaments (PHFs), and studies have shown that pathological double-helical fibers are mainly composed of hyperphosphorylated tau protein [23]. According to related reports, the number of NFTs in patient brains can be used as an important indicator for pathological determination of AD [24]. Abnormal phosphorylation of tau protein is an important pathological feature of AD. The study of abnormal phosphorylation of tau protein provides strong evidence for the pathogenesis of AD. Drug designing targeting tau protein has also been initiated. It has become an important starting point for the development of drugs for the treatment of AD. Therefore, studying neurodegenerative diseases from the perspective of tau protein pathology may have more general biological significance [26,27].

## 3. Deposition of β-Amyloid Protein (Aβ) in the Spinal Cord of AD Patients and Animal Models

Senile plaques formed by amyloid protein (Aβ) deposition in the brain are one of the most obvious pathological features of AD. Formation of senile plaques in AD patients is mostly caused by the release of Aβ around the cerebral vessels and then modified by various proteases, a process currently regarded as an irreversible pathological change [28,29]. The metabolism of Aβ is a dynamic process that is influenced by endogenous factors, such as genes, cells, and blood circulation, as well as exogenous factors, such as tissue hypoxia and stress. These combined factors influence the production, accumulation, release, and metabolism of Aβ [30]. Old age is an important cause of AD, which may be closely related to factors such as atrophy, poor blood circulation, and hypoxia in the brain tissue [31].

Despite the abundant results depicting the role of Aβ in brain degenerative diseases, brain trauma, and treatment, there are still few studies in the field of spinal cord injury which may be species-dependent. This may be partly ascribed to differences in the production and metabolism of amyloid precursor protein (APP) and Aβ in rats, mice, and humans [32], and the differences in the sensitivity and specificity of different antibodies targeting Aβ. Nevertheless, the expression of APP and Aβ after spinal cord injury (SCI) has begun to be studied, and the mechanism and roles of Aβ in inflammation, neuronal apoptosis, axonal regeneration, and myelination after SCI are being further studied [32]. 

Pathological studies have shown that Aβ deposition occurs in the dorsal horn of the gray matter and the central part of the white matter in the spinal cord of transgenic TgCRND8 mice [27]. Further research demonstrates that Aβ deposition in the spinal cord corresponds to the corticospinal tract as well as its projection area in TgCRND8 mice, suggesting that the sensorimotor cortex may represent the main source of Aβ, consistent with the theory that the sensorimotor cortex is particularly vulnerable during the development of AD [33]. Another study showed that a large number of Aβ aggregates was found in neurons of the spinal cord of 3-, 8-, and 11-month-old APPswe/PS1δ9 transgenic mice, but no extracellular amyloid plaques were detected [34]. The same pathological feature was noted in other AD transgenic mice [35,36,37]. Recently, Chu et al. [38] found that amyloid plaques start to appear at 11 weeks, in both gray and white spinal cord matter of the 5xFAD mouse, and exhibit a time dependent increase and differential distribution along the cord, with more plaques found in the cervix compared to other spinal segment levels [38]. Seo and colleagues [39] described in Tg2576 mice a wide range of and gradually worsening motor function deficits as early as 10 months of age, including a severe defect in the hind limb extension reflex and abnormal body trembling and hind limb tremors when suspended by the tail. Histological analyses showed severe degeneration of motor neurons in the spinal cord, including a severe reduction in the number of cholinergic neurons in the lumbar cord. In addition, high levels of *β*-amyloid accumulation were detected in the spinal cord in Tg2576 mice at the age of 16 months, accompanied with increased lipid peroxidation levels and significantly decreased mitochondrial metabolic activity. These changes could be partly suppressed by curcumin [39].

Verkkoniemi et al. [40] reported a variant AD (varAD), in an original Finnish family, which is distinct from classic AD because of the degeneration of the lateral corticospinal tracts. The authors histologically detected ‘‘cotton wool’’ plaques (CWPs) for Aβ42/43 but did not detect or only weakly detected Aβ40 isoforms of the amyloid-β peptide (Aβ). CWPs devoid of fibrillar amyloid cores were predominantly detected in the cerebral cortex, with the presence of non-neuritic amyloid plaques observed in the cerebellum. CWPs were particularly numerous in the medial motor cortex representing the lower extremities, and degeneration of the lateral corticospinal tracts was observed at the level of the medulla oblongata and the spinal cord [40]. Rudzinski et al. [41] for the first time described neuropathologic findings in early onset familial Alzheimer disease (EOFAD) owing to an N135S presenilin 1 (PSEN1) mutation. Patients presented with cognitive deficits, as well as less common features, such as spastic dysarthria, limb spasticity, and seizures. At autopsy, they had pathologic findings of AD, and histologic evidence of degeneration of the corticospinal tract [41].

An early study in patients with AD demonstrated that the amyloid plaques were distributed widely in the regions that were not affected in nondemented controls, with the spinal cord, dentate nucleus, and globus pallidus being sometimes (<50%) affected [42]. Guo et al. [23] did not detect amyloid deposition in the spinal cord tissue of AD patients in clinical studies. In an ‘amyloid only’ transgenic mouse model of AD, B6C3-Tg (APPswe,PSEN1dE9) 85 Dbo/Mmjax, in contrast to the abundant amyloid deposition detected in the cerebral cortices, hippocampus, olfactory bulbs, some white matter tracts and the cerebellar molecular layer, no amyloid aggregates were found in the midbrain, brainstem or spinal cord [43]. In summary, Aβ can accumulate in neurons in the spinal cord of AD mice, but a lack of amyloid plaque deposition is sometimes found in the spinal cord of some transgenic mice and AD patients, which may be determined by the pathological progression of AD. We thus hypothesize that some mechanisms that cause Aβ to deposit in the brain tissue may also contribute to subsequent plaque formation in the spinal cord tissue.

## 4. The Role of Inflammation in the Spinal Cord of AD Patients and Animal Models

More and more research has shown that AD is a chronic inflammatory process with severe immune and inflammatory responses in the brains of AD patients. Aβ deposition not only can lead to the formation of neuritic plaques (senile plaques) but also can function as an important inducer of inflammation that is characterized by activation of microglia and release of inflammatory factors to further damage neurons [44]. Researchers have shown that microglia only play a weak role in amyloid clearance, which is activated by amyloid deposits and release of inflammatory mediators, thus worsening the neurodegenerative conditions [45]. Although both morphological and genetic phenotypes of microglia in aged AD brain tissue suggest that they are activated, Streit and colleagues [46] detected a large number of aging and malnourished microglia. The decrease of neurotrophic factor secretion by microglia and impairment of phagocytosis with age, coupled with the increased secretion of inflammatory mediators, neuronal destruction, and amyloid aggregation, further promote the occurrence and development of AD.

Moreover, several studies have shown that the inflammatory response is closely related to the occurrence and development of AD [47,48,49]. Proliferation of glial cells can be observed in the hippocampus and cortex of AD mice, indicating that inflammation plays a role in the development and progression of AD. Wirth et al. [50] found that in APP/PS1 mice, the inflammatory response was not confined to the brain but also occurred in the spinal cord. Through immunohistochemical staining, they found that levels of GFAP and Iba-1 were upregulated in spinal cord tissue of 6-month-old APP/PS1 mice. Astrocytes and microglia were activated in the white matter of the spinal cord, which may help to understand the characteristics of axonal defects in this mouse model and to show that inflammatory processes are closely linked to axonal degeneration and loss of neurons [50]. Their findings further suggest that inflammatory responses not only occur in the brain, but also in the spinal cord tissue as pathological features of AD [47,48,49,50].

## 5. Other Pathological Changes in the Spinal Cord of AD Patients

Lewy bodies are morphologically concentric circular glass-like inclusion bodies commonly found in the substantia nigra, locus coeruleus, and the axons and cytoplasm of some cerebral cortical neurons in patients with neurodegenerative diseases [51]. Lewy neurites are positive for α-synuclein in both the neuropil and the white matter. At present, brain tissues in 30% to ~ 45% of AD patients show pathological changes of α-synuclein, mainly in the olfactory bulb, amygdala, and Meynert basal nucleus [51]. Associated pathological changes of α-synuclein were also found in the dorsal nucleus of the medullary vagus, the red nucleus in the brain, and the spinal cord of AD patients with Lewy bodies. In addition, small to moderate amounts of Lewy bodies and scattered Lewy neurites can be found in the mediolateral side of the lateral angle of the thoracic spinal cord and the anterolateral part of the gray matter of the sacral spinal cord of AD patients, thus playing a role in dyskinesia and autonomic dysfunction at the later stage of AD [51].

Some researchers believe that pathological changes associated with α-synuclein may originate in the lower part of the brainstem and then follow the ascending pathway to the upper brainstem and descending pathway into the spinal cord [52]. Bloch et al. observed that in AD patients, if there are pathological changes of α-synuclein in spinal cord tissue, related abnormal expression of α-synuclein occurs in the brain tissue [53]. However, there is no report showing the expression of α-synuclein exclusively in the spinal cord, suggesting a significant correlation between α-synuclein immunoreactivity and pathological changes in the brain tissue of AD patients. Further experiments indicate that, except for the medulla oblongata and olfactory bulb, the spinal cord may be the earliest, as well as the most susceptible part of the central nervous system (CNS) to α-synuclein-related pathological changes [53]. Although no apparent changes were found in the spinal cord of AD without Lewy bodies [54], severe neuron loss in the intermediolateral nucleus of the thoracic segment of the spinal cord was found in AD patients co-diagnosed with either PD or dementia with Lewy bodies [19]. It is of great necessity to identify how these Lewy body changes contribute to the severity of AD.

In addition, cytoplasmic tangle-shaped aggregates of small nuclear RNA (snRNA) were immunofluorescently found in post-mortem human spinal cord from both sporadic and familial AD cases, but not in other neurodegenerative disorders. snRNA was localized with tau and paired helical filaments, the main NTF component [55].

## 6. Summary and Prospective

AD is clinically manifested as progressive memory loss and cognitive dysfunction, which can seriously affect the daily life of patients and cause a serious economic burden to the family [56]. The exact pathogenesis of AD is still unclear, and commonly recognized points include heredity, environment, and living habits, and it still poses a major problem for scientific research [56].

**Figure 2:**
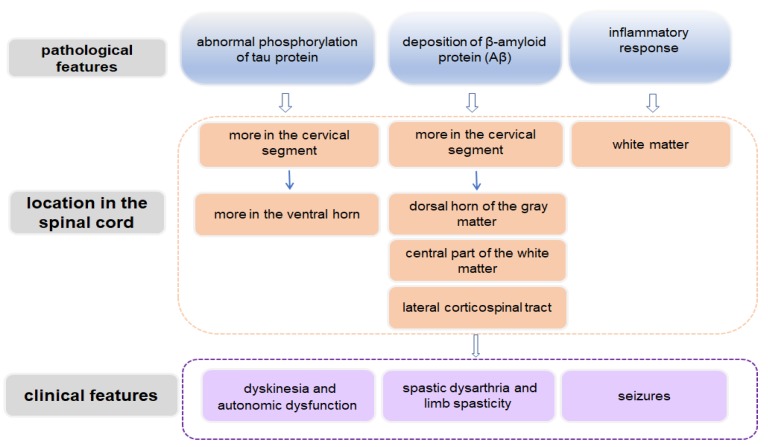
Schematic diagram demonstrating spinal cord-related pathological and clinical features in AD.

The typical pathological features of AD include extracellular Aβ deposition, intracellular NFTs caused by hyperphosphorylation of tau protein, massive neuronal loss, and reactive glial cell proliferation and activation [57]. These pathological features not only occur in brain tissue but also involve the spinal cord (Figure 2) [58,59]. In spite of this, related clinical reports about spinal cord changes in AD are limited, and some observations are based on AD models. However, it is not practical to prepare a mouse model displaying all the symptoms of AD. Since these mouse models show some pathological characteristics and symptoms in the spinal cord that are similar to those in human AD, it is of great value to use them to study the pathogenesis and treatment mechanism of AD.

In addition, it still needs to be elucidated whether these lesions represent pathological changes at the early stage of AD or further progression of the disease in the later stages, and how these spinal cord changes contribute to the severity of AD. Whether clinical symptoms and quality of life can be improved by ameliorating the spinal cord lesions in AD patients has yet to be further studied.

## References

[1] Wang Y.H., Ji Y. (2015). Development of alzheimer’s disease in the world. Chin. J. Modern Neurol. Dis..

[2] (2014). 2014 Alzheimer’s Disease Facts and Figures. Alzheimers Dement..

[3] Masoodi T.A., Al Shammari S.A., Al-Muammar M.N., Alhamdan A.A., Talluri V.R. (2013). Exploration of deleterious single nucleotide polymorphisms in late-onset Alzheimer disease susceptibility genes. Gene.

[4] Galante D., Corsaro A., Florio T., Vella S., Pagano A., Sbrana F., Vassalli M., Perico A., D’Arrigo C. (2012). Differential Toxicity, Conformation and Morphology of Typical Initial Aggregation States of Abeta1-42 and Abetapy3-42 Beta-Amyloids. Int. J. Biochem. Cell Biol..

[5] Mohsenzadegan M., Mirshafiey A. (2012). The immunopathogenic role of reactive oxygen species in Alzheimer disease. Iran. J. Allergy Asthma Immunol..

[6] Azizi G., Mirshafiey A. (2012). The potential role of proinflammatory and antiinflammatory cytokines in Alzheimer disease pathogenesis. Immunopharmacol. Immunotoxicol..

[7] Stefanova E., Pavlovic A., Jovanovic Z., Veselinovic N., Despotovic I., Stojkovic T., Sternic N., Kostic V. (2012). Vascular Risk Factors in Alzheimer’s Disease - Preliminary Report. J. Neurol. Sci..

[8] Lo R.Y., Jagust W.J., Initiative F.T.A.D.N. (2012). Vascular burden and Alzheimer disease pathologic progression. Neurol..

[9] Raudino F. (2016). Involvement of the Spinal Cord in the Alzheimer’s Disease: A Literature Review. Arch. Neurosci..

[10] Girgis S.I., Yates C.M., Fink G., MacIntyre I. (1990). Calcitonin gene-related peptide and calcitonin immunoreactivity in brain and spinal cord in Alzheimer-type dementia. J. Neurol. Sci..

[11] Yeh T.S., Ho Y.C., Hsu C.L., Pan S.L. (2018). Spinal cord injury and Alzheimer’s disease risk: A population-based, retrospective cohort study. Spinal Cord.

[12] Li Y.X., Cao M.X., Li Y.M., Zhang Q.Y., Shao H.R., Dong X.D. (2014). The Exploration between Tau Protein and Related Molecules in Alzheimer’s Disease. Med. Res. Educ..

[13] Botha H., Mantyh W.G., Graff-Radford J., Machulda M.M., Przybelski S.A., Wiste H.J., Senjem M.L., Parisi J.E., Petersen R.C., Murray M.E. (2018). Tau-negative amnestic dementia masquerading as Alzheimer disease dementia. Neurology.

[14] Ikezu T., Chen C., DeLeo A.M., Zeldich E., Fallin M.D., Kanaan N.M., Lunetta K.L., Abraham C.R., Logue M.W., Farrer L.A. (2018). Tau Phosphorylation is Impacted by Rare AKAP9 Mutations Associated with Alzheimer Disease in African Americans. J. Neuroimmune Pharmacol..

[15] Tiernan C.T., Mufson E.J., Kanaan N.M., E Counts S. (2018). Tau Oligomer Pathology in Nucleus Basalis Neurons During the Progression of Alzheimer Disease. J. Neuropathol. Exp. Neurol..

[16] Yamada M. (1978). On the Distribution of Senile Changes in the Spinal Cord. Psychiatry Clin. Neurosci..

[17] Saito Y., Murayama S. (2000). Expression of tau immunoreactivity in the spinal motor neurons of Alzheimer’s disease. Neurology.

[18] Dugger B.N., Hidalgo J.A., Chiarolanza G., Mariner M., Henry-Watson J., Sue L.I., Beach T.G. (2013). The Distribution of Phosphorylated Tau in Spinal Cords of Alzheimer’s Disease and Non-Demented Individuals. J. Alzheimers Dis..

[19] Zhu M., Wang L., Liu J., Gui Q., Guo Y., Hu Y., Zhang H. (2015). Histopathological and immunohistochemical study of spinal cord tissues in neurodegenerative diseases. Chin. J. Pathol..

[20] Parkkinen L., Soininen H., Alafuzoff I. (2003). Regional distribution of alpha-synuclein pathology in unimpaired aging and Alzheimer disease. J. Neuropathol. Exp. Neurol..

[21] Leroy K., Bretteville A., Schindowski K., Gilissen E., Authelet M., De Decker R., Yilmaz Z., Buée L., Brion J.-P. (2007). Early Axonopathy Preceding Neurofibrillary Tangles in Mutant Tau Transgenic Mice. Am. J. Pathol..

[22] Guo Y.J., Wang L.N., Zhu M.W., Zhang H.H., Hu Y.Z., Han Z.T., Li J.M., Wang D.X. (2011). Expression of tau-related protein in spinal cord of patients with Alzheimer’s disease. Chin. J. Pathol..

[23] Guo Y., Wang L., Zhu M., Zhang H., Hu Y., Han Z., Liu J., Zhao W., Wang D. (2016). Detection of Hyperphosphorylated Tau Protein and Alpha-Synuclein in Spinal Cord of Patients with Alzheimer’s Disease. Neuropsychiatr. Dis. Treat..

[24] Schmidt M.L., Zhukareva V., Perl D.P., Sheridan S.K., Schuck T., Lee V.M.-Y., Trojanowski J.Q. (2001). Spinal Cord Neurofibrillary Pathology in Alzheimer Disease and Guam Parkinsonism-Dementia Complex. J. Neuropathol. Exp. Neurol..

[25] Watson C., Paxinos G., Kayalioglu G., Heise C. (2009). Atlas of the Mouse Spinal Cord.

[26] Grundke-Iqbal I., Iqbal K., Tung Y.C., Quinlan M., Wisniewski H.M., Binder L.I. (1986). Abnormal phosphorylation of the microtubule-associated protein tau (tau) in Alzheimer cytoskeletal pathology. Proc. Natl. Acad. Sci. USA.

[27] Arriagada P.V., Marzloff K., Hyman B.T. (1992). Distribution of Alzheimer-Type Pathologic Changes in Nondemented Elderly Individuals Matches the Pattern in Alzheimer’s Disease. Neurology.

[28] A Carson J., Turner A.J. (2002). Beta-amyloid catabolism: Roles for neprilysin (NEP) and other metallopeptidases?. J. Neurochem..

[29] Nalivaeva N.N., Beckett C., Belyaev N.D., Turner A.J. (2012). Are Amyloid-Degrading Enzymes Viable Therapeutic Targets in Alzheimer’s Disease?. J. Neurochem..

[30] Fisk L., Nalivaeva N.N., Boyle J.P., Peers C.S., Turner A.J. (2007). Effects of Hypoxia and Oxidative Stress on Expression of Neprilysin in Human Neuroblastoma Cells and Rat Cortical Neurones and Astrocytes. Neurochem. Res..

[31] Dubrovskaia N.M., Nalivaeva N.N., A Plesneva S., A Feponova A., Turner A.J., A Zhuravin I. (2009). [Changes in the activity of amyloid-degrading metallopeptidases leads to disruption of memory in rats]. Журнал высшей нервнoй деятельнoсти им И П Павлoва.

[32] Bird S.M., Sohrabi H.R., Sutton T.A., Weinborn M., Rainey-Smith S.R., Brown B., Patterson L., Taddei K., Gupta V., Carruthers M. (2016). Cerebral Amyloid-Beta Accumulation and Deposition Following Traumatic Brain Injury--a Narrative Review and Meta-Analysis of Animal Studies. Neurosci. Biobehav. Rev..

[33] Yuan Q., Su H., Zhang Y., Chau W.H., Ng C.T., Song Y.Q., Huang J.D., Wu W., Lin Z.X. (2013). Amyloid Pathology in Spinal Cord of the Transgenic Alzheimer’s Disease Mice Is Correlated to the Corticospinal Tract Pathway. J. Alzheimers Dis..

[34] Li L.X., Zhang S.F., Zhang X., Le W.D. (2012). Autophagic Changes in Brain and Spinal Cord of Mice with Alzheimer’s Disease. J. Shanghai Jiaotong Univ..

[35] Pickford F., Masliah E., Britschgi M., Lucin K., Narasimhan R., Jaeger P.A., Small S., Spencer B., Rockenstein E., Levine B. (2008). The Autophagy-Related Protein Beclin 1 Shows Reduced Expression in Early Alzheimer Disease and Regulates Amyloid Beta Accumulation in Mice. J. Clin. Investig..

[36] Wirths O., Weis J., Szczygielski J., Multhaup G., Bayer T.A. (2006). Axonopathy in an APP/PS1 transgenic mouse model of Alzheimer’s disease. Acta Neuropathol..

[37] Jawhar S., Trawicka A., Jenneckens C., Bayer T.A., Wirths O. (2012). Motor Deficits, Neuron Loss, and Reduced Anxiety Coinciding with Axonal Degeneration and Intraneuronal Abeta Aggregation in the 5xfad Mouse Model of Alzheimer’s Disease. Neurobiol. Aging.

[38] Chu T.-H., Cummins K., Sparling J.S., Tsutsui S., Brideau C., Nilsson K.P.R., Joseph J.T., Stys P.K. (2017). Axonal and myelinic pathology in 5xFAD Alzheimer’s mouse spinal cord. PLoS ONE.

[39] Seo J.-S., Leem Y.-H., Kim S.-W., Lee K.-W., Han P.-L. (2010). Severe Motor Neuron Degeneration in the Spinal Cord of the Tg2576 Mouse Model of Alzheimer Disease. J. Alzheimer’s Dis..

[40] Verkkoniemi A., Kalimo H., Paetau A., Somer M., Iwatsubo T., Hardy J., Haltia M. (2001). Variant Alzheimer Disease With Spastic paraparesis: Neuropathological phenotype. J. Neuropathol. Exp. Neurol..

[41] Rudzinski L.A., Fletcher R.M., Dickson D.W., Crook R., Hutton M.L., Adamson J., Graff-Radford N.R. (2008). Early Onset Alzheimer’s Disease with Spastic Paraparesis, Dysarthria and Seizures and N135S Mutation in PSEN1. Alzheimer Dis. Assoc. Disord..

[42] Ogomori K., Kitamoto T., Tateishi J., Sato Y., Suetsugu M., Abe M. (1989). Beta-Protein Amyloid Is Widely Distributed in the Central Nervous System of Patients with Alzheimer’s Disease. Am. J. Pathol..

[43] Finnie G.S., Gunnarsson R., Manavis J., Blumbergs P.C., Mander K.A., Edwards S., Van den Heuvel C., Finnie J.W. (2017). Characterization of an ‘Amyloid Only’ Transgenic (B6c3-Tg(Appswe,Psen1de9)85dbo/Mmjax) Mouse Model of Alzheimer’s Disease. J. Comp. Pathol..

[44] Akiyama H., Barger S., Barnum S., Bradt B., Bauer J., Cole G.M., Cooper N.R., Eikelenboom P., Emmerling M., Fiebich B.L. (2000). Inflammation and Alzheimer’s disease. Neurobiol. Aging.

[45] Lucin K.M., Wyss-Coray T. (2009). Immune Activation in Brain Aging and Neurodegeneration: Too Much or Too Little?. Neuron.

[46] Streit W.J. (2005). Microglia and Neuroprotection: Implications for Alzheimer’s Disease. Brain Res. Rev..

[47] Goetzl E.J., Schwartz J.B., Abner E.L., Jicha G.A., Kapogiannis D. (2018). High complement levels in astrocyte-derived exosomes of Alzheimer disease. Ann. Neurol..

[48] Qi Y., Klyubin I., Cuello A.C., Rowan M.J. (2018). Nlrp3-Dependent Synaptic Plasticity Deficit in an Alzheimer’s Disease Amyloidosis Model in Vivo. Neurobiol. Dis..

[49] Torika N., Asraf K., Apte R.N., Fleisher-Berkovich S. (2018). Candesartan Ameliorates Brain Inflammation Associated with Alzheimer’s Disease. CNS Neurosci. Ther..

[50] Wirths O., Breyhan H., Marcello A., Cotel M.C., Bruck W., Bayer T.A. (2010). Inflammatory Changes Are Tightly Associated with Neurodegeneration in the Brain and Spinal Cord of the App/Ps1ki Mouse Model of Alzheimer’s Disease. Neurobiol. Aging.

[51] Guo Y.J., Wang L.N., Zhu M.W., Zhang H.H., Hu Y.Z., Han Z.T., Zhao W.Q., Wang D.X. (2010). Expression of A-Synuclein in the Spinal Cord of Alzheimer’s Disease. Chin. J. Contemp. Neurol. Neurosurg..

[52] Saito Y., Kawashima A., Ruberu N.N., Fujiwara H., Koyama S., Sawabe M., Arai T., Nagura H., Yamanouchi H., Hasegawa M. (2003). Accumulation of phosphorylated alpha-synuclein in aging human brain. J. Neuropathol. Exp. Neurol..

[53] Bloch A., Probst A., Bissig H., Adams H., Tolnay M. (2006). alpha-Synuclein pathology of the spinal and peripheral autonomic nervous system in neurologically unimpaired elderly subjects. Neuropathol. Appl. Neurobiol..

[54] Beach T.G., Adler C.H., Sue L.I., Vedders L., Lue L., White Iii C.L., Akiyama H., Caviness J.N., Shill H.A., Sabbagh M.N. (2010). Arizona Parkinson’s Disease Consortium. Multi-organ distribution of phosphorylated alpha-synuclein histopathology in subjects with Lewy body disorders. Acta Neuropathol..

[55] Hales C.M., Dammer E.B., Diner I., Yi H., Seyfried N.T., Gearing M., Glass J.D., Montine T.J., Levey A.I., Lah J.J. (2014). Aggregates of Small Nuclear Ribonucleic Acids (snRNAs) in Alzheimer’s Disease. Brain Pathol..

[56] Villemagne V.L., Dore V., Burnham S.C., Masters C.L., Rowe C.C. (2018). Imaging Tau and Amyloid-Beta Proteinopathies in Alzheimer Disease and Other Conditions. Nat. Rev. Neurol..

[57] Djogo N., Jakovcevski I., Muller C., Lee H.J., Xu J.C., Jakovcevski M., Kugler S., Loers G., Schachner M. (2013). Adhesion Molecule L1 Binds to Amyloid Beta and Reduces Alzheimer’s Disease Pathology in Mice. Neurobiol. Dis..

[58] Gibbons G.S., A Banks R., Kim B., Changolkar L., Riddle D.M., Leight S.N., Irwin D.J., Trojanowski J.Q., Lee V.M.Y. (2018). Detection of Alzheimer Disease (AD)-Specific Tau Pathology in AD and NonAD Tauopathies by Immunohistochemistry With Novel Conformation-Selective Tau Antibodies. J. Neuropathol. Exp. Neurol..

[59] Quiroz Y.T., Sperling R.A., Norton D.J., Baena A., Arboleda-Velásquez J.F., Cosio D., Schultz A., LaPoint M., Guzman-Velez E., Miller J.B. (2018). Association Between Amyloid and Tau Accumulation in Young Adults With Autosomal Dominant Alzheimer Disease. JAMA Neurol..

